# Characterization of Bacterial Etiologic Agents of Biofilm Formation in Medical Devices in Critical Care Setup

**DOI:** 10.1155/2012/945805

**Published:** 2012-01-24

**Authors:** Sangita Revdiwala, Bhaumesh M. Rajdev, Summaiya Mulla

**Affiliations:** ^1^Department of Microbiology, Government Medical College, Surat, Veer Narmad South Gujarat University, Surat 395001, India; ^2^Department of Forensic Medicine, Government Medical College, Surat, Veer Narmad South Gujarat University, Surat 395001, India

## Abstract

*Background*. Biofilms contaminate catheters, ventilators, and medical implants; they act as a source of disease for humans, animals, and plants. *Aim*. Critical care units of any healthcare institute follow various interventional strategies with use of medical devices for the management of critical cases. Bacteria contaminate medical devices and form biofilms. *Material and Methods*. The study was carried out on 100 positive bacteriological cultures of medical devices which were inserted in hospitalized patients. The bacterial isolates were processed as per microtitre plate. All the isolates were subjected to antibiotic susceptibility testing by VITEK 2 compact automated systems. *Results*. Out of the total 100 bacterial isolates tested, 88 of them were biofilm formers. A 16–20-hour incubation period was found to be optimum for biofilm development. 85% isolates were multidrug resistants and different mechanisms of bacterial drug resistance like ESBL, carbapenemase, and MRSA were found among isolates. *Conclusion*. Availability of nutrition in the form of glucose enhances the biofilm formation by bacteria. Time and availability of glucose are important factors for assessment of biofilm progress. It is an alarm for those who are associated with invasive procedures and indwelling medical devices especially in patients with low immunity.

## 1. Introduction

Microorganisms universally attach to surfaces and produce extracellular polysaccharides, resulting in the formation of a biofilm. Biofilms pose a serious problem for public health because of the increased resistance of biofilm-associated organisms to antimicrobial agents and the potential for these organisms to cause infections in patients with indwelling medical devices. An appreciation of the role of biofilms in infection should enhance the clinical decision-making process. Many bloodstream infections and urinary tract infections are associated with indwelling medical devices and, therefore, are (in most cases) biofilm associated. The most effective strategy for treating these infections may be removal of the biofilm contaminated device [1].

When an indwelling medical device is contaminated with microorganisms, several variables determine whether a biofilm develops. First the microorganisms must adhere to the exposed surfaces of the device long enough to become irreversibly attached. The rate of cell attachment depends on the number and types of cells in the liquid to which the device is exposed, the flow rate of liquid through the device, and the physicochemical characteristics of the surface. Components in the liquid may alter the surface properties and also affect the rate of attachment. Once these cells irreversibly attach and produce extracellular polysaccharides to develop a biofilm, rate of growth is influenced by flow rate, nutrient composition of the medium, antimicrobial-drug concentration, and ambient temperature [2].

There are many works that discuss some features of biofilm-positive bacteria, but there is no consistency in the conditions which are feasible for biofilm formation among authors [3–7]. The only agreement is in the culture temperature, 37°C seems to be appropriate. Other conditions, for example, presence of nutrition and time of cultivation, vary in many publications. In our study we paid attention to those culture conditions that differ in most authors. We investigated the potential relationship between colonization of different medical devices by various clinical bacterial isolates and to determine the differences in biofilm formation in different conditions and to determine the minimum time and conditions necessary for the development of a homogenous and mature biofilm layer [3].

## 2. Materials and Methods

Approval was obtained from our institutional review board. The study was carried out on 100 positive bacteriological cultures of medical devices which were inserted in hospitalized patients.

**Catheter Culture Technique**

All catheters/devices submitted to the clinical laboratory for culture during a 3-year period were studied. Each catheter coming to the clinical laboratory for culture was directly cultured by roll plate method then placed in 10 mL of tryptic soya broth (Himedia, Mumbai, India), incubated for 2 hrs at 37°C and then vortexed for 15 seconds. Broth was then surface-plated by using a wire loop on Blood agar, Chocolate agar, and MacConkey agar (Himedia, Mumbai, India) [8].

Isolates derived later from the clinical laboratory for the purpose of our study were frozen in nutrient broth with 15% glycerol at −20°C. Samples retrieved for the study were grown on blood agar plates and were processed as described below.

Cultures retrieved from the frozen material retained the same biochemical reactions, confirming that no alteration had occurred in bacterial isolate because of storage and processing.

## 3. Biofilm Formation and Quantification of Activity against Biofilms

**Preparation of Inoculum**

3 different media were taken: tryptic soya broth, tryptic soya broth with 0.25% glucose, and tryptic soya broth with 0.5% glucose for culture. Isolated colonies were inoculated and incubated for 24 hrs in these media then cultures were diluted 1 : 200 with respective fresh media.

**Control**

Biofilm-producing reference strains of *Acinetobacter baumannii* (ATCC 19606) and *Pseudomonas aeruginosa* (ATCC 27853) and nonbiofilm forming reference strains of *Staphylococcus aureus* (ATCC 25923) and *E. coli* (ATCC 25922) were used [9].

**Microtitre Plate Assay**

Biofilm formation was induced in 96-well flat-bottomed polystyrene microtitre plates. An aliquot of 200 *μ*L of diluted bacterial suspension was added to each well and incubated for 16 h, 20 h, and 24 h at 37°C. At the end of incubation period, the wells were carefully aspirated and washed twice with 300 *μ*L of phosphate-buffered saline (PBS, pH, 7.2) to remove planktonic bacteria. Wells were emptied and dried before biomass quantification of the biofilms was performed by staining. The staining was done with 200 *μ*L of 0.1% safranine and 0.1% crystal violet into respective wells for 45 minutes. At the end of time, the wells were carefully washed twice with distilled water to remove excess stain. After staining, 200 *μ*L ethanol/acetone (90 : 10) was added to each well to dissolve remaining stain from the wells. The optical density was then recorded at 492 nm with 630 nm reference filter using an ELISA reader [3, 10–13].

Wells originally containing uninoculated medium, nonbiofilm producing bacteria and known biofilm producing bacteria were used as controls for cutoff, negative controls, and positive controls, respectively. The test was carried out in quadruplicate, results were averaged and standard deviations were calculated.

The cutoff was defined as three standard deviations above the mean ODc [14]. Each isolate was classified as follows: weak biofilm producer OD = 2 × ODc, moderate biofilm producer 2 × ODc < OD = 4 × ODc, or strong biofilm producer OD > 4 × ODc [9, 15].

Antimicrobial susceptibility testing was performed by using VITEK 2 compact automated system according to the norms of Clinical Laboratory Standards Institute (CLSI). Relevant statistical analysis was done.

## 4. Results

The demographic profile of the patients under study indicates 41% female and 59% male patients with bacteriological positive culture. Medical ICU: 36 (44%) was the predominant source of specimen followed by surgery ward: 18 (22%) and neonatal ICU: 16 (20%), least from obstetrics and gynecology ward and pediatrics ward: 6 (7% each). 59 endotracheal tubes (ETT), 11 CVC (central vascular catheter) tips, 10 Foley's catheter tips, 7 abdominal drain tubes, 5 nephrostomy tubes, 4 tracheostomy tubes, 3 D. J. (Double J) stent tip, and 1 SPC (supra pubic catheter) tip were found bacteriologically positive under study group. Bacteriological profile of group showed 23% *Acinetobacter baumannii, *23% *Pseudomonas aeruginosa, *20% *Klebsiella pneumonia sub spp. pneumoniae, *16% *E. coli, *9% coagulase negative *Staphylococci, *4% *Enterobacter cloacae, *3% *Enterococci, *and 2% *Staphylococcus aureus *isolates.  Table 1 shows that in endotracheal tube colonization by *Acinetobacter, Pseudomonas *and *Klebsiella *as prevalent bacterial isolates, followed by *E. coli*. Present study showed that frequently isolated bacteria in central venous line (CVP tip) were Coagulase negative *staphylococci *(46%) followed by *Acinetobacter *(18%), *P. aeruginosa *(18%), *Enterococci *species (9%), and *S. aureus *(9%)*. Enterococci *are more commonly associated with colonization of central venous lines and Foley's catheter.

**Figure 1:**
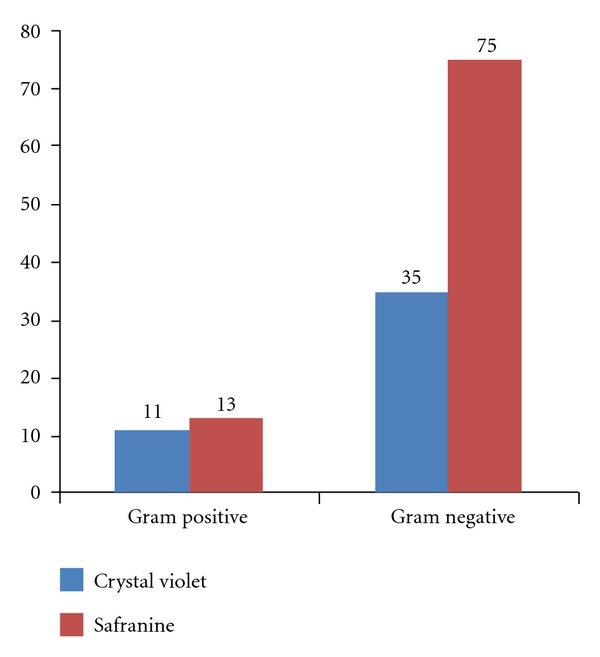
Showing ability of safranine and crystal violet staining methods to detect biofilms by microtitre plate assay.

Out of 100 clinical isolates tested, 88 were found to be biofilm formers by micro titer plate method. Out of two different staining methods; 0.1% safranine had detected 88 biofilm producers while 0.1% crystal violet had detected 69 biofilm producers (See Figure 1).

Biofilm formation in response to different concentrations of glucose was studied. Tryptic soya broth without glucose showed biofilm formation in 75 (85%) isolates. Out of 75, 2 were strong and 28 were moderate biofilm formers as shown in Table 3. In tryptic soya broth with 0.25% glucose; 81 (92%) were found positive, of which 3 were strong and 30 were moderate biofilm formers. In tryptic soya broth with 0.5% glucose; 67 (76%) were found positive, out of which 4 were strong and 28 were moderate biofilm formers.

Biofilm formation at different incubation time periods was studied. At 16 hr incubation period; 88 (100%) were found to be positive, out of it, 3 were strong and 28 were moderate biofilm formers. At 20 hr incubation period, 81 (92%) found positive, 2 were strong and 36 were moderate biofilm formers. At 24 hr incubation period; 76 (86%) found positive, 4 were strong, and 29 were moderate biofilm formers.

Table 4 shows antimicrobial drug resistance profile of bacterial isolates suggesting majority as multiple drug resistant. Phenotypic evaluation showing expression of different drug-resistance mechanisms includes ESBL production (23%), carbapenemase production (34%), AmpC production (7%), carbapenem impermeability (41%), and modification of PBP (13%) responsible for resistance among betalactam antibiotics tested. Drug resistance by Van A (35%), Van B (35%), and TEC (50%) was seen among glycopeptides antibiotics. For MLSB (macrolide lincosamide streptogramin B) group; constitutive (87%) and inducible (1%) have both mechanisms worked for resistance.

## 5. Discussion

Indwelling medical devices are frequently used in all health setup while critical care units of hospitals use multiple medical devices for treatment and intervention in patient care. Endotracheal tube amounted to more than 50% of our specimen; these may be due to more specimens from patients admitted in critical care which were either intubated or needing ventilator support in multispecialty hospital. Second most common specimen for investigation was central venous catheters that amounted to 12% of total specimen volume under study. Central venous catheters (CVCs) pose a greater risk of device-related infection than does any other indwelling medical device, with infection rates of 3 to 5%. Catheters may be inserted for administration of fluids, blood products, medications, nutritional solutions, and hemodynamic monitoring. 12% of the specimen was urinary catheter for our study. Urinary catheters were used for many indications in hospital like to measure urine output, collect urine during surgery, prevent urinary retention, or control urinary incontinence.

These organisms isolated in this study may originate from the skin of patients or healthcare workers, tap water to which entry ports are exposed, or other sources in the environment [2]. *Acinetobacter*, *Pseudomonas, Klebsiella, Staphylococcus, Enterobacter, *and* E. coli* are the most common causes of nosocomial infections, and that may be common cause of colonization in indwelling medical devices even responsible for biofilm production [10, 11]. These microorganisms survive in hospital environments despite unfavorable conditions such as desiccation, nutrient starvation, and antimicrobial treatments. It is hypothesized that their ability to persist in these environments, as well as their virulence, is a result of their capacity to colonize medical devices [8].

In a study by Feldman et al. [16], it was documented that the interior of the ETT of patients undergoing mechanical ventilation rapidly became colonized with gram-negative microorganisms which commonly appeared to survive within a biofilm. While it appears that colonization of the ETT may begin from as early as 12 h, it is most abundant at 96 h.

Colonization of the ETT with microorganisms commonly causing nosocomial pneumonia appears to persist in many cases despite apparently successful treatment of the previous pneumonia. A study by Donlan et al. showed that the organisms most commonly isolated from central venous catheter biofilms are *Staphylococcus epidermidis, S. aureus, Candida albicans, P. aeruginosa, K. pneumoniae, *and *Enterococcus faecalis *[9, 10]. Stickler et al. [17] showed that the organisms commonly contaminating this urinary catheter and developing biofilms are *S. epidermidis, Enterococcus faecalis, E. coli, Proteus mirabilis, P. aeruginosa, K. pneumoniae, *and other gram-negative organisms [2, 9–11]. The study of different mechanisms of drug resistance showed isolates commonly found positive for ESBL, carbapenemase production in gram-negative organism and MRSA, vancomycin resistance among gram-positive organisms. Resistant strains are circulating in the environment of the hospital and are responsible for contamination/colonization of different indwelling medical devices used for patient management and complicate the course of treatment.

Indwelling medical devices are frequently used in all health setup while critical care units of hospitals use multiple medical devices for treatment and intervention in patient care. Endotracheal tube amounting to more than 50% of our specimen; may be due to the fact that more specimens are from patients admitted in critical care which were either incubated or needing ventilator support in multispecialty hospital. The second most common specimen for investigation was central venous catheters amounting 12% of total specimen volume under study. Central venous catheters (CVCs) pose a greater risk of device-related infection than does any other indwelling medical device, with infection rates of 3% to 5%. Catheters may be inserted for administration of fluids, blood products, medications, nutritional solutions, and hemodynamic monitoring. 12% specimen was of urinary catheter for our study. Urinary catheter were used for many indications in the hospital like to measure urine output, collect urine during surgery, prevent urinary retention, or control urinary incontinence.

In a study by Feldman et al. [16], it was documented that the interior of the ETT of patients undergoing mechanical ventilation rapidly became colonized with gram-negative microorganisms which commonly appeared to survive within a biofilm. While it appears that colonization of the ETT may begin from as early as 12 h, it is most abundant at 96 h. Colonization of the ETT with microorganisms commonly causing nosocomial pneumonia appears to persist in many cases despite apparently successful treatment of the previous pneumonia. A study by Donlan et al. showed that the organisms most commonly isolated from central venous catheter biofilms are *Staphylococcus epidermidis, S. aureus, Candida albicans, P. aeruginosa, K. pneumoniae, *and *Enterococcus faecalis* [6, 12]. Stickler et al. [17] showed that the organisms commonly contaminating this urinary catheter and developing biofilms are *S. epidermidis, Enterococcus faecalis, E. coli, Proteus mirabilis, P. aeruginosa, K. pneumoniae, *and other gram-negative organisms [6]. One study by Rao et al. showed 30% biofilm forming bacterial isolates among medical devices like endotracheal tubes followed by central venous catheters and urinary catheters are third most common site of biofilm forming bacterial colonization [9].

## 6. Conclusion

Out of the two different staining methods, safranine 0.1% and crystal violet 0.1%, safranine staining gave more positive, stable, and accurate results in terms of reproducibility, for both, gram-positive as well as gram-negative bacteria. 20 hr incubation time was found to be optimum for detection of biofilms produced by bacteria. Moderate to weak biofilm producing bacteria although do attach to the surfaces, but detachment occurs early because of weak binding. Strong biofilm producers can be detected even at 24 hours of incubation period. Availability of nutrition favors biofilm formation by bacteria so glucose enhances biofilm forming ability of bacteria, but effect of osmolarity and pH cannot be ruled out on biofilm formation.

ESKAPE' group (Enterococci, Staphylococcus aureus, Klebsiella, Acinetobacter, Pseudomonas, and Enterobacter cloacae) of bacteria that are important nosocomial treats in ICUs; which are biofilm producers and responsible for chronic and multidrug-resistant infections. There is presence of multidrug-resistant isolates in the environment of hospital and majority of them were biofilm producers, so it is an alarm for those who are associated with invasive procedures and indwelling medical devices especially in patients with low immunity. They are responsible for increased morbidity and mortality under hospital environment and impacts are major on patient outcome. Biofilm bacteria exhibit various mechanisms of drug resistance transfer so spread of drug resistance among ICU infection is a major threat to patient care in critical care units of health care institutes.

## Figures and Tables

**Table 1:** Relation of clinical bacterial isolates and the type of device inserted.

	*Acinetobacter baumannii*	*Pseudomonas aeruginosa*	*Klebsiella pneumonia sub spp. Pneumonia*	*E. coli*	*Enterobacter cloacae*	*Coagulase negative staphylococci*	*Enterococci*	*Staphylococcus aureus*
Endotracheal tube	16	17	13	7	3	1	1	1
CVP tip	2	0	2	0	0	5	1	1
Foley's catheter tip	1	3	1	3	1	0	1	0
Abdominal drain tube	1	1	0	3	0	2	0	0
Nephrostomy tube	2	0	2	0	0	1	0	0
Tracheostomy tube	1	2	1	0	0	0	0	0
D.J. stent tip	0	0	1	2	0	0	0	0
SPC tip	0	0	0	1	0	0	0	0

Total	23	23	20	16	4	9	3	2

**Table 2:** Quantitative analysis of biofilm production by clinical bacterial isolates as evaluated by microtitre plate method.

	Strong	Moderate	Weak
*Acinetobacter baumannii*	1	16	5
*Pseudomonas aeruginosa*	2	8	8
*Klebsiella pneumonia sub spp. Pneumonia*	1	8	11
*E. coli*	0	1	10
*Enterobacter cloacae*	0	2	2
*Coagulase negative staphylococci*	1	5	2
*Enterococci*	0	2	1
*Staphylococcus aureus*	0	1	1

Total	5	44	39

**Table 3:** Screening of 100 bacterial isolates for biofilm formation by microtitre plate method in different media and at 16, 20, and 24 hr incubation periods.

	No. of isolates								
Biofilm formation (OD_492–630 mm_)	TSB			TSB, 0.25% glucose			TSB, 0.5% glucose		
	16 hr	20 hr	24 hr	16 hr	20 hr	24 hr	16 hr	20 hr	24 hr
High (ODc **< **OD** > **2 × ODc)	1	1	1	1	2	1	2	1	2
Moderate (2 × ODc < OD = 4 × ODc)	17	24	19	19	18	17	15	21	17
Weak (ODc** < **OD** > **2 × ODc)	39	43	44	44	49	43	33	32	30

Experiment was done in quadruplet and repeated two times. All OD_492–630 mm_ values were expressed as average with standard deviation.

**Table 4:** Different mechanisms of drug resistance in isolates of indwelling medical devices.

Name of bacteria	ESBL	Carbapenemase	Alteration of PBP	Van A/B
*Acinetobacter baumannii*	15	25		
*Pseudomonas aeruginosa*	25	30		
*Klebsiella pneumoniae sub spp. Pneumoniae*	30	30		
*Escherichia coli*	25	15		
*Coagulase negative Staphylococci*			40	0
*Enterobacter cloacae*	5	0		
*Enterococci spp.*				45
*Staphylococcus aureus*			60	55
